# The Current Role of Circulating Cell-Free DNA in the Management of Hepatocellular Carcinoma

**DOI:** 10.3390/cancers17061042

**Published:** 2025-03-20

**Authors:** Alkistis Papatheodoridi, Vasileios Lekakis, Antonios Chatzigeorgiou, George Papatheodoridis

**Affiliations:** 1 Department of Clinical Therapeutics, Medical School of National and Kapodistrian University of Athens, “Alexandra” General Hospital of Athens, 11528 Athens, Greece; alkistispapath@gmail.com; 2 First Department of Gastroenterology, Medical School of National and Kapodistrian University of Athens, General Hospital of Athens “Laiko”, 11527 Athens, Greece; lekakis.vas@gmail.com; 3 Department of Physiology, Medical School of National and Kapodistrian University of Athens, 11527 Athens, Greece; achatzig@med.uoa.gr

**Keywords:** hepatocellular carcinoma, cell-free DNA, tumor DNA, methylation

## Abstract

Circulating cell-free DNA (cfDNA) has emerged as a compelling candidate of liquid biopsy markers for the diagnosis and prognosis of several cancers. We systematically reviewed data on the role of cfDNA markers in the diagnosis, prognosis and treatment of hepatocellular carcinoma (HCC). Early studies suggested that levels of circulating cfDNA, mitochondrial DNA and cfDNA integrity are higher in patients with HCC than chronic liver diseases. In subsequent studies, methylation changes in circulating tumor DNA (ctDNA) as well as cfDNA fragmentation patterns and circulating nucleosomes were found to offer high sensitivity (>60%) and excellent specificity (>90%) for HCC diagnosis. The predictive role of cfDNA markers and ctDNA has been assessed in a few studies including untreated patients with HCC providing promising results for prediction of survival. However, port-hepatectomy detection of cfDNA/ctDNA markers or copy number variation indicators of cfDNA seem to reflect minimum residual disease and thus a high risk for HCC recurrence. The same markers can be useful for prediction after transarterial chemoembolization, radiofrequency ablation, radiotherapy and even systemic therapies. In conclusion, cfDNA markers can be useful in HCC surveillance, improving early diagnosis rates, as well as for monitoring treatment effectiveness and minimal residual disease post-treatment.

## Simple Summary

Circulating cell-free DNA (cfDNA) has emerged as a compelling candidate of liquid biopsy markers for the early diagnosis and prognosis of several cancers. We systematically reviewed the existing data on the potential role of cfDNA markers in the diagnosis, prognosis and treatment of hepatocellular carcinoma (HCC). According to our findings, there are many data showing that specific cfDNA species could be rather useful liquid biomarkers for early HCC diagnosis, although further research and methodological improvements are necessary. Moreover, cfDNA markers can be useful for monitoring treatment effectiveness and for early detection of minimal residual disease post-treatment, thus optimizing patients’ management.

## 1. Introduction

Liver cancer currently represents the sixth most prevalent cancer and the third leading cause of cancer-related death worldwide, with hepatocellular carcinoma (HCC) being responsible for 75–85% of such cases [1,2]. Not only has the incidence of HCC been rising over recent years, but the projections for the future are more discouraging, with HCC-related deaths expected to continuously increase over the next decade [3]. Therefore, early diagnosis, accurate prediction and optimized treatment of HCC are of great clinical importance.

HCC usually develops in patients with pre-existing chronic liver disease, more frequently chronic infection with hepatitis B virus (HBV) and especially cirrhosis, cirrhosis due to chronic infection with hepatitis C virus (HCV), alcohol-related cirrhosis and any other cause of cirrhosis. In recent years, advanced liver injury or cirrhosis due to metabolic dysfunction-associated steatotic liver disease (MASLD) is becoming the most common risk factor for HCC in most Western countries [4,5]. All patients at increased risk of HCC are recommended to undergo surveillance for early HCC diagnosis [4]. Although the HCC diagnosis can often be made by non-invasive imaging methods such as computed or magnetic resonance tomography [4], the current methods of HCC surveillance, including 6-monthly ultrasonography with or without alfa fetoprotein (AFP) measurements, remain suboptimal for several reasons. Thus, HCC is often diagnosed at advanced stages, leading to limited treatment options and reduced patient survival. In addition, there are no markers of accurate prediction or, most importantly, markers which may tailor and optimize treatment efforts [6].

Recently, liquid biopsy has been a field of growing interest, especially in oncology, as it is a non-invasive method which can be potentially helpful for the identification of specific cancer biomarkers and characteristics [6]. In particular, circulating cell-free DNA (cfDNA) has emerged as a compelling candidate of liquid biopsy markers [7]. Hence, our systematic review focuses on the potential role of cfDNA markers in the diagnosis, prognosis and treatment of HCC.

## 2. Circulating cfDNA

**Figure 1:**
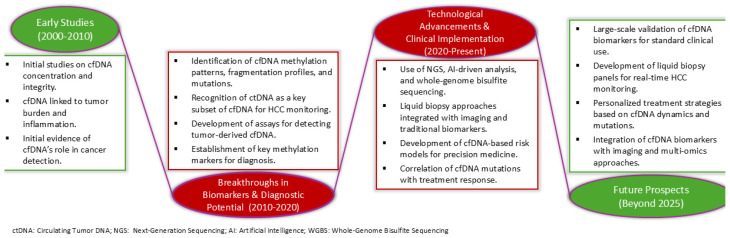
Progression of cell-free DNA (cfDNA) research in hepatocellular carcinoma (HCC): key milestones and future perspectives.

Over recent years, there has been a lot of interest in the research of cfDNA in HCC. The major advancements and anticipated developments in this field are visually presented in Figure 1.

### 2.1. Types and Subtypes

cfDNA usually presenting as circulating double-stranded DNA fragments associated with nucleosomes is released into the bloodstream as a result of apoptosis, necrosis and secretion. DNA can also be transferred by extracellular vehicles including exosomes [8]. Multiple studies have explored cfDNA as a potential surrogate marker for risk stratification of a plethora of diseases including inflammatory and autoimmune diseases and cancers. In patients with cancer, tumor cells release specific components of cfDNA which constitute the circulating tumor DNA (ctDNA) representing <1% to 90% of the total cfDNA [6,7]. The pool of serum cfDNA includes circulating fragments of nuclear gene-coding and non-coding DNA, as well as other sequences such as Alu repeat sequences and mitochondrial DNA, collectively termed DNA species. Moreover, epigenetic modifications—primarily DNA methylation—have been linked to a wide range of human diseases, from neurological and psychiatric disorders to tumorigenesis. Herein, we summarize the methodological detection of aforementioned cfDNA parameters and their utilization in the field of HCC diagnosis and monitoring.

### 2.2. Methods of Detection

Several features of the cfDNA have been employed to develop assays that could be used as a readout for the diagnosis, prognosis and therapy of HCC. Specifically, approaches pertinent to the total quantity and integrity of cfDNA, alterations in its methylation status, DNA fragmentation, as well as detection of mutations have been used in this field [9].

Firstly, estimation of total cfDNA concentration and integrity are considered traditional methods in HCC research and semi-quantitative or quantitative PCR approaches for the detection of specific housekeeping genes or ALU amplicons are utilized to this direction. Other methods, such as Qubit Fluorometric Quantification for the detection of double-stranded DNA, are also used; nevertheless, the heterogeneity of HCC may nowadays dispute their importance during HCC prediction and monitoring [9,10,11].

Global cfDNA methylation or analysis of the methylation status of CpG islands of specific genes such as HOXA1, EMX1, TSPYL5, SEPT9, ECE1, PFKP and CLEC11A have been identified and utilized as indicators of the progression and prognosis of HCC [6]. The methodological detection in this case includes a spectrum of approaches including targeted low-cost ones such as methylation-specific PCR assays and pyrosequencing approaches focused in specific genomic regions and genes, up to more sophisticated NGS-based approaches such as whole-genome bisulfite sequencing [12]. As far as DNA fragmentation of cfDNA is concerned, the base pair size of the “fragments” depends on nucleosome packing and is used predominantly in the case of patients at risk for developing HCC [6]. Whole-genome sequencing approaches are usually applied for fragmentation analyses, and the short/long fragment ratio serves as an indicator of fragmentation [13]. In addition, mutations at the promoters or other regions of genes such as *TP53*, *TERT* and *CTNNB1* have been used for this purpose, especially in the case of early-stage HCC. In most cases, commercially available kits developed for the isolation of circulating nucleic acids are used, followed by PCR enhancement of the fragments of interest, which are then analyzed using several sequencing platforms. The Digital Polymerase Chain Reaction (dPCR) system and Tagged-amplicon deep sequencing (TAm-Seq) have also been used for the same purpose [6,14,15,16]. More expensive untargeted approaches, such as whole-exome sequencing, have also been utilized in certain studies [9].

## 3. Search Strategy, Selection Criteria and Data Extraction

From January 2000 to November 2024, PubMed was searched to identify all medical literature included under the following search text terms: “cell free DNA” AND “hepatocellular carcinoma”. In addition, a manual search of relevant review articles and of the retrieved original studies was performed.

All studies published in English as full papers were included, if they fulfilled the following criteria: (1) observational studies (case–control, cross-sectional or cohort) or randomized trials; (2) included at least 50 patients for HCC diagnosis and prognosis or at least 30 patients for HCC therapy; (3) assessed the role of cfDNA in the diagnosis, prognosis or therapy of patients with HCC.

**Figure 2:**
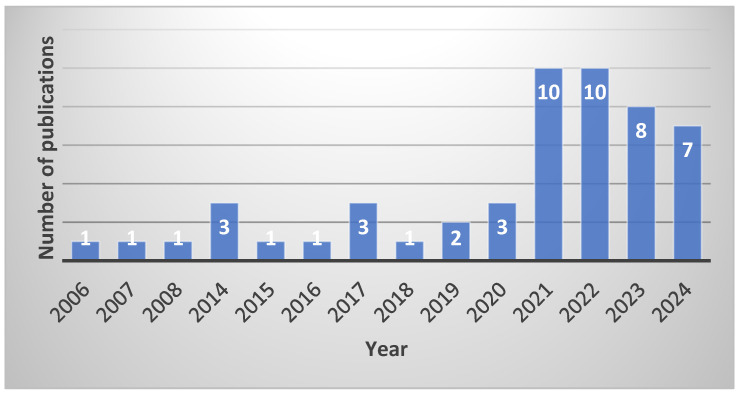
Cell-free DNA research in hepatocellular carcinoma over the years.

A literature search was performed by two independent reviewers (AP, VL), who determined which studies could be potentially included. Two lists of selected papers were compared for concordance, and discrepancies were discussed and arbitrated by a third reviewer (GP). Each study in the list of selected papers was evaluated by two independent reviewers (AP, VL) to determine whether it fulfilled all the inclusion criteria. These two reviewers extracted data from the selected papers according to a predefined form. The two data summary tables were compared for concordance, and discrepancies were discussed and arbitrated by a third reviewer (GP). The interest in cfDNA research on HCC as reflected by the annual number of publications is shown in Figure 2.

## 4. cfDNA for HCC Diagnosis

**Figure 3:**
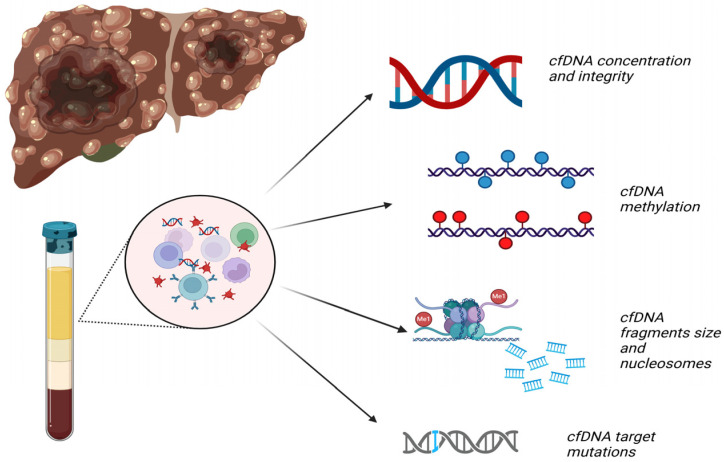
Cell-free DNA (cfDNA) species used for diagnosis of hepatocellular carcinoma. Created in BioRender, Papatheodoridi A (2025).

cfDNA concentration and several cfDNA species, such as integrity, methylation and target mutations, have been assessed as potential markers of HCC diagnosis (Figure 3), but the results sometimes seem to vary among different studies [17]. The key findings of the studies in this setting are described below and are presented in Table 1, Table 2, Table 3 and Table 4.

### 4.1. cfDNA Concentration and Integrity (Table 1)

In three early studies from Japan, serum cfDNA levels were found to be higher in patients with HCV-related HCC compared to HCV carriers or healthy controls [18,19,20], suggesting however that serum cfDNA level in such cases may be associated with the inflammatory tumor status [19]. In a more recent study from Egypt, cfDNA levels were also reported to be useful in discriminating patients with HCV cirrhosis from patients with HCV-related HCC [21]. Finally, one study from China indicated that the tumor content of circulating cfDNA is related to the development of HBV-related HCC and presents an increasing trend during the 4-year period preceding clinical diagnosis of HCC, whereas it is correlated with tumor burden and worse survival [22].

Our group has shown that cfDNA integrity is higher in chronic hepatitis B (CHB) patients with than those without HCC and that increased cfDNA integrity is related to worse one-year HCC prognosis [10]. In addition, we have reported that the levels of RNAse P in cfDNA, an indicator of amplifiable genomic DNA, are increased in the serum of CHB patients even 5 years before the diagnosis of HCC [25]. In the previously reported study from Egypt, levels of cfDNA integrity (Alu 247/Alu 115) were shown to be higher in HCV patients with HCC than HCV patients with cirrhosis [21], while, in another study from Egypt, cfDNA integrity was reported to be lower in the plasma of HCV genotype 4 patients with HCC than HCV genotype 4 patients with cirrhosis [26]. In a study from China, plasma cfDNA integrity was found to be lower in patients with liver cancers (including HCC, intrahepatic cholangiocarcinoma and liver metastasis from other primary tumors) compared to a small sample of individuals with benign liver diseases and healthy controls [24]. On the other hand, in a study from Hong Kong, elevated amounts of mitochondrial DNA were detected in the plasma of patients with HCC compared to CHB patients with or without cirrhosis and healthy controls [23].

### 4.2. cfDNA Methylation in HCC Diagnosis (Table 2)

Numerous studies have supported the potential role of cfDNA methylation in HCC diagnosis. In 2017, in a large study from China including 1098 HCC cases and 835 healthy controls divided into training and validation cohorts, epigenetic alterations in cfDNA and specifically a circulating tumor DNA (ctDNA) methylation marker were reported to offer excellent diagnostic accuracy (AUROC: 0.94–0.97) with high specificity (90–94%) and sensitivity (83–86%), which was further correlated with tumor burden, stage and treatment response [27]. Along the same line, additional studies from China have suggested that several diagnostic models based on methylation markers and mutations can exhibit high rates of sensitivity (60–94%) and specificity (91–98.5%) for HCC diagnosis [28,29,30,31,32], whereas the combined use of traditional markers including AFP appears to potentially increase the diagnostic performance of such tests [28,30]. Moreover, cfDNA methylation ratio [methylation copies/(methylation copies plus unmethylation copies)] was also suggested to have good diagnostic accuracy for HCC diagnosis [33]. In a case–control study from Germany and the USA, a DNA-methylation panel established by next-generation sequencing (NGS) offered very good diagnostic accuracy with excellent specificity (97%) and acceptable sensitivity (58%), which was improved after the combined use of the NGS panel with AFP (AUROC: 0.90) [34]. In a phase 2 prospective clinical trial, HelioLiver Test, which combined cfDNA methylation patterns with clinical characteristics and protein tumor markers, showed sensitivity of 85% for any or early stage (76%) of HCC diagnosis performing better than AFP and GALAD score and having similarly high specificity (>90%) [35].

**Table 2:** Circulating cell-free DNA (cfDNA) methylation markers or models for diagnosis of hepatocellular carcinoma (HCC).

First Author, Year [Reference]		HCC Pts	Controls	Species of cfDNA	Sensitivity	Specificity	AUROC (95% CI)	Other Key Findings
Xu, 2017 [27]	Training cohort	715	560	HCC-specific methylation marker Panel by targeted bisulfite sequencing	86%	94%	0.97 (0.96–0.98)	Correlation with tumor burden, stage and treatment response; prediction of survival
	Validation cohort	383	275		83%	90.5%	0.94 (0.93−0.96)	
Wang, 2020 [33]		97	80 and 46 CHB/CHC	cfDNA methylation ratio [methylation cp/(methyla-tion + unmethylation cp)]	79%	89%	0.81 (0.72–0.90)	
Lewin, 2021 [34]	Training cohort	41	46 LC	cfDNA methylation markers: HCCBloodTest (Epigenomics AG) and NGS panel	77% and 57%; NGS and AFP: 68%	64% and 97%; NGS and AFP: 97%	NGS: 0.85 (0.78–0.91) NGS and AFP: 0.90 (0.84–0.95)	
	Testing cohort	60	103 LC					
Luo, 2022 [28]	Training cohort	120	290 (65 HBsAg+) and 92 LC	cfDNA methylation profiles based on tissue methylation profiles from pts and controls	86%	98%	0.98 (0.97–0.99)	For early-stage HCC diagnosis: AUROC 0.93 (95% CI: 0.90–0.96)
	Validation cohort	67	242 (56 HBsAg+) and 111 LC		84%	96%	0.97 (0.95–0.99)	
Lin, 2022 [35]	Phase 2 study	122	125 CLD	HelioLiver Test: methylation, clinical and tumor markers	85%	91%	0.94 (0.92–0.97)	HelioLiver Test superior sensitivity for HCC detection than AFP and GALAD score
Wang, 2022 [29]	Training cohorts	30 and 60	30 and 60	(Epi)Genetic alterations in cfDNA and genome-wide discovery of methylation markers	93%	95%	0.96 (0.93–1.00)	
	Independent cohort	58	198		90%	94%	0.93 (0.90–0.97)	
Phan, 2022 [30]	Testing cohort	58	121 LC or CH	cfDNA methylation markers (450 target regions, 18,000 CpG sites)	62%	91%	0.84 (0.82–0.90)	Plus GALAD score—AUROC: 0.87 (95% CI: 0.85–0.94) (sensitivity: 69%, specificity: 96%)
	Validation cohort	48	72 LC or CH		60%	96%	0.84 (0.82–0.90)	
Deng, 2023 [31]		62	39 and 67 CLD	cfDNA methylation by whole-genome sequencing plus deep learning techniques	94% (early stage HCC: 90%)	98.5% (early stage HCC: 89.5%)	0.99 (0.98–0.99)	Superior diagnostic accuracy than AFP
Guo, 2023 [32]		73	84 and 22 CLD	cfDNA methylation by enzymatic methyl sequencing	90%	97%	0.96 (0.93–0.99)	
Han, 2014 [36]		160 HBV	133 (88 CHB)	Methylation of TGR5 promoter	TGR5+ AFP: 65–81%	TGR5 + AFP: 85–39%	TGR5 without AFP: 0.67 (0.61–0.73.)	
Li, 2014 [37]		136 HBV	35 and 46 CHB	Methylation at IGFBP7 promoter	65%	83%	0.74	HCC with vascular invasion: higher IGFBP7 methylation rates (84% vs. 60%, *p* = 0.010)
Huang, 2014 [38]		66	43 CLD	Methylation at INK4A promoter	65/39/20% for 5/7/10% CpG	87/96.5/99% for 5/7/10% CpG cut-off	0.82	INK4A methylation and AFP: sensitivity 80% (45.5% for AFP alone)
Oussalah, 2018 [39]	Initial study	51	135 LC	mSEPT9 test: SEPT9 promoter methylation in cfDNA	94%	84%	0.94 (0.90–0.97)	
	Replication	47	56 LC		85%	91%	0.93 (0.86–0.97)	
Kim, 2023 [40]		313	413 (211 high risk)	Methylation markers of RNF135 and LDHB	57%; and AFP: 70%	94%; and AFP: 93%	0.80 (0.76–0.83)	Superior sensitivity than AFP alone (45%)
Cai, 2019 [41]		1204	958 and 392 CHB/LC	Genome-wide 5-hydro-xymethylcytosines: 32-gene diagnostic model	83%	76%	0.88 (0.86–0.91) Early stage HCC: 0.85 (0.81–0.89)	Superior performance than AFP alone
Cai, 2021 [42]	Training set	103	167	HCC score: 5-hydroxy-xymethylcytosine signatures and AFP and des-γ-carboxy-prothrombin	79%	91%	0.92 (0.88−0.92)	Prediction of relapse and survival after resection in high HCC recurrence risk pts
	Test set	32	60		94%	78%	0.95 (0.89−0.95)	
Guo, 2024 [43]	Training cohort	293	266 (96 CHB/LC)	Differentially methylated regions (DMRs) by NGS and quantitative methylation-specific PCR HepaAiQ: 20 best DMRs	86%	92%	0.94 (0.93–0.96)	High postoperative HepaAiQ score: higher HCC recurrence risk (Hazard Ratio: 3.33, *p* < 0.001)
	Validation cohort	205	318 (100 CHB/LC)		84%	90%	0.94 (0.93–0.95)	
	Independent cohort	65	124 CHB/LC		71%	90%		
Kim, 2024 [44]		36	213 HIV	Inflammation-DNAm score (54 CpGs)			0.94 (0.90–0.98)	

AFP: alfa fetoprotein; AUROC: area under receiving operating characteristic; CH: chronic hepatitis; CHB: chronic hepatitis B; CI: confidence interval; CLD: chronic liver disease; GALAD score: serum AFP, AFP-L3, des-γ-carboxy-prothrombin, gender, age; HBV: hepatitis B virus; HIV: human immunodeficiency virus; IGFBP7: insulin-like growth factor-binding protein 7; LC: liver cirrhosis; LDHB: Lactate Dehydrogenase B; NGS: next-generation sequencing; PCR: polymerase chain reaction; pts: patients; RNF135: Ring Finger Protein 135.

More specific methylation markers have also been evaluated. In 2014, three studies from China reported that hypermethylation of specific cfDNA regions (TGR5 promoter, IGFBP7 promoter, INK4A promoter) are detected more frequently in HCC cases compared to controls often including CHB patients, whereas the combined use of AFP seemed to improve the diagnostic accuracy of these markers, especially sensitivity [36,37,38]. In 2018, in two phase 2 studies, the methylation of SEPT9 promoter in cfDNA was shown to offer high sensitivity (85–94%) and specificity (84–91%) in discriminating HCC cases among cirrhotic patients [39]. Recently, in a study from Korea, specific HCC methylation markers, including Ring Finger Protein 135 and Lactate Dehydrogenase B, were also shown to offer good sensitivity (57%) for HCC diagnosis, especially in combination with AFP (sensitivity 70%), maintaining excellent specificity (93–94%) [40]. Additionally, studies from China suggested that liquid biopsy based on 5-hydroxymethylcytosine signatures of cfDNA may accurately distinguish HCC patients from healthy controls and high HCC risk patients [41,42], whereas the diagnostic accuracy can increase by combining other HCC biomarkers such as AFP and des-gamma-carboxy prothrombin [42]. In 2024, a large multicenter study including three cohorts reported that HCC-specific differentially methylated regions (DMRs) by NGS and quantitative methylation-specific polymerase chain reaction (PCR) could offer a model based on DNRs that may represent an effective tool for HCC detection and prognosis, demonstrating high sensitivity (71–86%) and specificity (90–92%) for HCC diagnosis [43]. Finally, in a study including 249 HIV patients, inflammation-related DNA methylation signatures of cfDNA were shown to be associated with an increased risk of developing HCC [44].

### 4.3. cfDNA Fragments Size and Nucleosomes (Table 3)

Recent research indicated that cfDNA fragmentation patterns and circulating nucleosomes hold potential as diagnostic biomarkers for HCC [45]. In several studies from China, cfDNA fragmentation profiles (e.g., fragment size, tumor fraction, copy number and 4-mer end motifs) determined by low-coverage whole-genome sequencing sometimes combined with machine learning programs were shown to offer excellent sensitivity (87–97%) and specificity (80–99%) for the detection of HCC [13,46,47]. In another study from China, the combination of copy number variations and cfDNA fragment size with AFP was reported to result in improved sensitivity of 75% and specificity of 98% for diagnosing HCC [48]. Similar results were presented when a different cfDNA fragmentation profile using low-coverage whole-genome sequencing was analyzed with a machine learning approach and tested in a training USA/European cohort and then validated in a Hong Kong cohort [49]. Moreover, in a study from Vietnam and the USA, cfDNA fragmentomics resulted in 13 HCC-related genes, which seemed to offer high sensitivity and specificity for HCC diagnosis [50].

**Table 3:** Circulating cell-free DNA (cfDNA) fragmentation profiles and nucleosomes for diagnosis of hepatocellular carcinoma (HCC).

First Author, Year [Reference]		HCC Pts	Controls	Species of cfDNA	Sensitivity	Specificity	AUROC (95% CI)	Other Key Findings
Jin, 2021 [46]		197 HBV	187 HBV	Fragment size, tumor fraction, copy number and 4-mer end motifs	NA	NA	NA	These markers can help in HCC detection
Meng, 2021 [48]		76	247	Copy numbers and fragment size plus AFP	75%	98%	0.95	High score: shorter recurrence-free survival
Chen, 2021 [51]	Training	255	260 and 347 LC	HIFI score = 4 cfDNA genomic features: nucleosome footprint, motif, 5hmC, fragmentation profiles				
	Validation	95	100 and 100 LC		96%	95%	0.995 (0.99–1.000)	
	Test	131	116 and 1800 LC		95%	98%	0.996 (0.992–0.999)	
Sun, 2022 [13]		110 HCC (105 HBV, 5 HCV)	342 (100 HBV and 99 HBV LC)	Fragment size by whole-genome sequencing	87%	88%	NA	
Zhang, 2022 [47]	Training cohort	159 (and 26/7 ICC/mixed)	170 (51 LC/HBV)	cfDNA fragmentomic profiles using whole-genome sequencings	97%	99%	0.995	
	Test cohort	157 (and 26/6 ICC/mixed)	164 (51 LC/HBV)		NA	NA	NA	
Fan, 2023 [52]	Training cohort	47	1706 LC	aMAP2 Plus score, aMAP score and AFP and 3 cfDNA signatures (nucleosome, fragment and motif scores)	70%	92%	0.89 (0.83–0.94)	
	Validation cohort	67	2520 LC		67%	88%	0.85 (0.80–0.90)	
Foda, 2023 [49]	Training cohort	75	426 (133 CLD)	cfDNA fragmentation profiles by low-coverage whole-genome sequencing and machine learning program	Average/High risk: 88%/85%	Average/High risk: 98%/80%	Average/High risk: 0.98 (0.97–0.99), /0.90 (0.86–0.94)	
	Validation cohort	90	133 (101 LC/HBV)		NA	NA	High risk: 0.97 (0.95–0.99)	
Nguyen, 2023 [50]	Test cohort	55	55	ctDNA fragmentomics, 13 HCC-related gene mutations	89%	82%	0.88	Incorporation of mutation fragment length enhances early HCC detection
	Validation	54	53		81%	81%	0.86	
Chen, 2024 [53]	Stage 1	510	4561 LC	PreCar Score = 5 cfDNA genomic features: nucleo-some footprint, motif, 5hmC, fragmentation profiles	94%	95%	NA	PreCar Score: higher sensitivity than US or AFP; PreCar Score plus US: improved sensitivity for early/very early HCC
	Stage 2	76	2487 LC		51%	96%	0.79 (0.73–0.85)	

AFP: alfa fetoprotein; AUROC: area under receiving operating characteristic; CI: confidence interval; CLD: chronic liver disease; HBV: hepatitis B virus; ICC: intrahepatic cholangiocarcinoma; LC: liver cirrhosis; pts: patients; US: ultrasonography.

Another group from China created a score, the HIFI score, which comprised nucleosomes, 5-end motif, 5hmC and fragment size patterns assessed by NGS and tested its performance in a large training and validation cohort including 508 HCC patients, 2250 patients with cirrhosis and 476 healthy controls, reporting high accuracy in detecting HCC [51]. In 2024, the same group presented data for an updated version of the HIFI score, which also included cfDNA copy number variation and was named PreCar. The score was tested in a multi-center, large-scale, cross-sectional study of two cohorts including, collectively, 586 HCC patients and 7048 cirrhotic patients and showed high sensitivity and specificity in diagnosing HCC patients [53]. Finally, another study from China proposed scores which incorporated cfDNA fragmentation, motif scores and nucleosomes with an established HCC risk score (aMAP) and AFP achieving excellent diagnostic accuracy for HCC diagnosis (AUROC: 0.85–0.89) with good sensitivity (67–70%) and excellent specificity (88–92%) in both the training and validation cohorts [52].

### 4.4. cfDNA Target Mutations (Table 4)

A few studies have assessed the role of cfDNA mutations in HCC diagnosis, sometimes in combination with known HCC serum markers. In particular, in another study from China, three specific mutations in cfDNA, namely TERT, TP53 and CTNNB1, were detected by Circulating Single-Molecule Amplification and Resequencing Technology (cSMART). The combination of these mutations with established HCC serum markers, specifically AFP, AFP-L3 and PIVKA-II, resulted in excellent accuracy (AUROC: 0.87) with improved sensitivity and specificity for early HCC diagnosis [54]. Using another approach, virus host chimera DNA was also assessed as a potential biomarker using NGS and was reported to have a sensitivity of 98% for HCC detection [55].

Overall, the various cfDNA detection and characterization methods used for HCC diagnosis not only share different specificities and sensitivities, but also lack implementation in larger, multicenter studies. In addition, other approaches within the field of liquid biopsy, such as exosomes or circulating tumor cells, may be utilized in the panel of HCC detection in the near future. Overall, the research of cfDNA and liquid biopsy in HCC needs to be focused on large multicenter studies and the extraction of algorithms for the utilization of the most appropriate methods for HCC diagnosis.

**Table 4:** Circulating cell-free DNA (cfDNA) target mutations for diagnosis of hepatocellular carcinoma (HCC).

First Author, Year [Reference]		HCC Pts	Controls	Species of cfDNA	Sensitivity	Specificity	Other Key Findings
Wu, 2023 [54]	Test cohort	151	145 LC	Gene mutation signatures by cSMART and NGS: TERT, TP53 and CTNNB1 muta-tions plus serum markers	89%	81%	
	Validation cohort	112	88 LC		81%	82%	
Li, 2020 [55]		50 HBV		Virus–host chimera DNA (vh-DNA)	98% (detection limit: 1.5 cm)	NA	Correlation between vh-DNA copy number and tumor size: r = 0.7955, *p* < 0.0001.
Campani, 2024 [56]		173	56 CLD	ctDNA and cfDNA: mutations in TERT, TP53, CTNNB1, PIK3CA and NFE2L2			ctDNA mutations correlated with active HCC (40.2%) vs. controls (1.8%).

AUROC: area under receiving operating characteristic; CI: confidence interval; CLD: chronic liver disease; cSMART: Circulating Single-Molecule Amplification and Resequencing Technology; ctDNA: cell tumor DNA; HBV: hepatitis B virus; LC: liver cirrhosis; pts: patients.

## 5. cfDNA for HCC Prognosis

The role of cfDNA markers in the prognosis of untreated patients with HCC has been evaluated in only two studies, both of which assessed their prognostic role after HCC treatment as well (Table 5).

In a retrospective case–control study from China and the USA, the prognostic role of cell tumor DNA (ctDNA) methylation markers was explored in a large cohort of 1098 HCC patients [27]. A panel of eight methylation indicators, which were correlated with survival outcomes, were integrated into a prognostic prediction model and verified in independent training (*n* = 680) and validation cohorts (*n* = 369). The combined prognosis score (cp-score) efficiently categorized patients into high risk and low risk, with high-risk cases having independently reduced survival [HR: 2.4 (95% CI: 1.9–3.1) in the training cohort and 1.5 (95% CI: 1.2–1.9) in the validation cohort]. Moreover, the integration of cp-score with TNM staging enhanced prognosis accuracy, yielding superior AUC values (0.79 and 0.76 for training and validation datasets) in comparison to TNM staging alone. The study also emphasized the dynamic characteristics of ctDNA methylation alterations, illustrating its effectiveness in tracking treatment response. Patients who underwent full tumor excision demonstrated diminished cp-scores post-surgery, whereas individuals with tumor growth or recurrence displayed elevated values.

A recently published retrospective case–control cohort study from China involving patients with HBV-related HCC emphasized the predictive value of tumor-derived circulating cfDNA [22]. High tumor content in cfDNA was found to correlate with advanced tumor stage (*p* < 0.001) and poorer survival (HR: 12.3, 95% CI: 1.4–107.9; *p* = 0.023). In addition, paired pre- and post-treatment samples from 17 patients indicated a substantial correlation between tumor content in cfDNA and tumor burden as well as disease progression. In advanced-stage HCC, the sensitivity of cfDNA tumor content for identifying active disease was 82% for Barcelona Clinic Liver Cancer (BCLC) B and 95% for BCLC C, indicating its capacity to monitor tumor dynamics post-treatment.

## 6. cfDNA and HCC Therapy

The role of cfDNA markers in the prediction of HCC patients after treatment has been evaluated in several studies, mostly including cases treated with surgical or locoregional therapies and only a few including cases treated with systemic therapies [57].

### 6.1. Surgical or Locoregional Therapies (Table 6)

In an early case–control study from Japan, the predictive significance of cfDNA levels post-curative hepatectomy was assessed in a cohort of 87 patients with HCV-related HCC and found to be correlated with clinical outcomes [20]. High cfDNA levels (≥117.8 ng/mL) were independently associated with reduced overall survival (HR: 3.4; 95% CI: 1.5–7.6, *p* = 0.004) and increased probability of extrahepatic recurrence (HR: 4.5; 95% CI: 1.3–14.9, *p* = 0.014).

The predictive role of postoperative circulating cfDNA levels was also explored in another prospective cohort study from China including 82 HCC patients who underwent curative hepatectomy, with postoperative cfDNA levels quantified by a fluorometric dsDNA assay [58]. Patients with high (>2.95 ng/μL) compared to low cfDNA levels had significantly lower recurrence-free survival (RFS) (median RFS: 14 months vs. 19.5 months, *p* = 0.022). Multivariate analysis indicated that postoperative cfDNA (HR: 1.3, 95% CI: 1.1–1.6, *p* = 0.023), tumor count and microvascular invasion (MVI) serve as independent predictors of recurrence. Furthermore, postoperative cfDNA levels exhibited a correlation with significant clinical characteristics, including tumor diameter, vascular invasion and advanced BCLC stages.

The prognostic role of copy number variations (CNVs) in circulating cfDNA at multiple levels (genome-wide, chromosomal-arm and bin level) was investigated in a prospective cohort study from China including 117 patients with HCC who were treated with surgical resection or radiofrequency ablation (RFA) [59]. A significant agreement in CNV profiles was noted between cfDNA and tumor tissue DNA, with sensitivity and specificity >70% at both the bin and chromosomal-arm levels, suggesting cfDNA as a reliable surrogate for tissue-based genomic analysis. High values of three genome-wide CNV indicators (tumor fraction, prediction score and stability score) were associated with poorer overall survival (OS) and RFS (HR for OS: 3.7–4.0). Additionally, the chromosomal-arm-level analysis revealed that high-frequency CNVs at chromosomal-arm levels were associated with worse OS and RFS, while combined indicators from specific chromosomal arms (e.g., 8q gain with 17p loss) may improve further the prognostic prediction. Finally, a novel risk score was developed using significant CNVs at the bin (1 Mb) level. Patients exhibiting elevated bin scores experienced markedly inferior OS and RFS, with AUCs for the bin score of 0.82 and 0.75 for 1- and 3-year survival, respectively, indicating superior prognostic accuracy compared to other CNV indicators.

**Table 6:** The main characteristics of studies evaluating the role of cell-free DNA (cfDNA) on response/prognosis after surgical or locoregional therapy for hepatocellular carcinoma (HCC).

First Author, Year [Reference]	Study Population	Main Objective	Marker Type	Methodology	Key Findings
Tokuhisa, 2007 [20]	96 HCV-HCC patients (87 resection) and 100 HCV carriers	cfDNA levels for prediction of survival and distant metastasis	cfDNA concentration	Real-time PCR quantification of cfDNA	**Prognostic cutoff**—High cfDNA levels (>117.8 ng/mL): shorter OS (HR: 3.4, 95% CI: 1.5–7.6, *p* = 0.004) and greater risk of EHR (HR: 4.5, 95% CI: 1.3–14.9, *p* = 0.014). **Tumor characteristics**—cfDNA levels positively associated with tumor size and differentiation.
Long, 2020 [58]	82 HCC patients after hepatectomy	Postoperative cfDNA levels as biomarker for recurrence and prognosis in HCC patients	Postoperative cfDNA concentra-tions	cfDNA postoperatively using a fluorometric dsDNA assay	**Postoperative cfDNA cutoff for recurrence:** 2.95 ng/μL (AUC: 0.68, sensitivity: 88%, specificity: 45%). **Survival analysis**—High postoperative cfDNA (>2.95 ng/μL): poorer RFS (median 14 vs. 19.5 mos, *p* = 0.02). **Independent risk factors for recurrence:** cfDNA (HR: 1.287, *p* = 0.023), tumor number (HR: 0.037, *p* = 0.004) and microvascular invasion (HR: 0.127, *p* = 0.005).
Wang, 2021 [59]	117 HBV-related HCC patients receiving radical treatments	Multi-level cfDNA CNV indicators for prognosis after radical treatments	cfDNA CNVs (TFx, P-score, S-score)	Low-coverage whole-genome sequencing of plasma cfDNA, CNV profiling at genome-wide, chromosomal-arm and bin levels	**Genome-wide CNVs**—Three genome-wide indicators (TFx, P-score and S-score): associated with poorer RFS and OS; High TFx (≥0.02), P-score (≥0.74) and S-score (≥0.04): associated with worse prognosis. **Chromosomal-arm CNVs—17p loss/8q gain:** HR 4.31/3.20 for death (*p* < 0.001) and HR 2.74/2.49 for recurrence (*p* ≤ 0.003). **Bin-level CNVs**—A novel bin score (1 Mb resolution): outperformed genome-wide and chromosomal-arm indicators in prognosis (AUC: 0.820 for 1-year survival and 0.746 for 3-year survival).
Fu, 2022 [60]	258 HCC patients undergoing curative liver resection	Preoperative ctDNA for early recurrence prediction	ctDNA	Blood samples collected preoperatively, ctDNA detection and mutation analysis, RNA sequencing for immune profiling	**Early recurrence prediction**—Number of ctDNA-mutant genes: associated with early HCC recurrence (HR: 2.2, *p* < 0.001). **High-risk patients**—Mutations in HRGs (APC, ARID1A, CDKN2A, FAT1, LRP1B, MAP3K1, PREX2, TERT, TP53): worse RFS (HR: 13, *p* < 0.001). **Prognostic nomogram**—Combination of ctDNA risk level and TNM stage predicted recurrence with high accuracy (C index: 0.76). **Therapy response prediction**—FAT1 or LRP1B but no TP53 mutations: worse PFS with lenvatinib plus ICIs after recurrence (HR: 17, *p* < 0.001). **Immune profiling**—ctDNA status correlates with tumor immune infiltration.
Dong, 2022 [61]	64 HCC patients treated with TACE, 57 LC patients and 32 healthy controls	cfDNA copy number profiling and TFx as biomarkers for TACE efficacy	cfDNA, CNV, TFx	LD-WGS of cfDNA pre- and post-TACE; tumor fraction and CNV profiling	**Pre-TACE**—High TFx (≥0.1): correlation with tumor burden and prediction of shorter PFS (97 vs. 189 days) and OS (243 vs. 630 days). **Post-TACE**—Reductions in TFx (>0.03): better PFS (163 vs. 63 days, *p* = 0.007) and aligned with imaging-based assessments. **Lipiodol deposition**—Amplifications in chromosomes 1q,3p, 6p, 8q, 10p,12q, 18p and18q were associated with poor lipiodol deposition. **TFx** outperformed AFP levels in predicting tumor burden and therapeutic outcomes (Sensitivity: 85.3%, Specificity: 94.4%).
Muraoka, 2021 [15]	67 HCC patients: 32 TACE, 35 TKIs	cfDNA hTERT promoter mutations for predicting responses	cfDNA (hTERT promoter mutation)	cfDNA by dPCR; analysis of mutant vs. wild-type cfDNA changes	**TACE**—Mutant cfDNA rate increased post-TACE (33% to 73%, (*p* = 0.001). Post-TACE correlations: mutant cfDNA changes with tumor necrosis (*p* < 0.001) and wild-type cfDNA changes with AST changes (*p* < 0.001). **TKIs**—Mutant cfDNA levels peaked within 1 week only in responders, who had longer PFS (10 vs. 3.4 months, *p* = 0.004).
Nakatsuka, 2021 [62]	100 HCC patients: TACE: 32, MTAs (lenvatinib, sorafenib, regorafenib): 35, RFA: 33	cfDNA levels and mutation profiles for tumor response and treatment outcomes	cfDNA, ctDNA, TERT promoter mutations	cfDNA levels measured pre- and post-treatment; TERT mutations detected using ddPCR; ultra-deep sequencing (22,000× coverage)	**Baseline cfDNA**—High (>70.7 ng/mL) vs. low baseline cfDNA: shorter OS (5.5 vs. 14 mos, *p* < 0.001). **Post-treatment**—cfDNA levels increased post-TACE (49 to 249 ng/mL, *p* < 0.001) and post-RFA (39 to 96 ng/mL, *p* < 0.001); rate of TERT mutations increased post-TACE (45% to 57%) and post-RFA (42% to 55%). **Post-MTA**—cfDNA levels increased after initiation of MTA; >1.5-fold cfDNA increase within 1 week: longer PFS (10 vs. 3.4 mos, *p* = 0.004). **Lenvatinib response**: Associated with mutations in genes like AMER1, MLL3 and NOTCH2 identified by ultra-deep sequencing.
Li, 2022 [63]	60 HCC patients treated with primary liver resection	TP53 mutations in exosomal cfDNA and HCC prognosis and treatment response	TP53 mutationstatus	Exosome extraction; TP53 mutations detected using ddPCR	High- vs. low-frequency mutations (MD/TD ≥67% vs. <67%): shorter RFS (68 vs. 368 days, *p* < 0.01). High-frequency mutation: poor prognosis, though patients with better pathological characteristics (HR: 4.61; *p* = 0.003).
Kim, 2023 [64]	37 patients with advanced HCC under-going RT	cfDNA for prediction of treatment response in advanced HCC treated with RT	cfDNA genomic instability score (I-score)	cfDNA analysis at pre-RT and 1 week post-RT, whole-genome sequencing, genomic instability scoring	**Genomic instability**—I-score: predictive of PFS (AUC = 0.71; sensitivity = 50%, specificity = 91%). **Pre-RT I-score**—Pre-treatment I-score (≥6.3) was associated with worse PFS (HR = 2.69, *p* = 0.017) and correlated with tumor burden. **Post-RT I-score**—High I-score (≥6.2): poor responses (non-complete response, *p* = 0.034). **Dynamic changes in I-score**—Delta I-score ratio reflected treatment effects, with negative/positive ratios in responders/non-responders.
Campani, 2024 [56]	173 HCC patients and 56 controls (including cirrhotic patients)	ctDNA as biomarker for tumor biology and treatment monitoring	ctDNA	NGS on MiSeq and droplet based digital PCR for TERT, TP53, CTNNB1, PIK3CA NFE2L2 mutations	**ctDNA mutations**—40% of active HCC, 14.6% of inactive HCC and 1.8% of controls. - Increasing prevalence in advanced stages (BCLC C: 65% vs. BCLC 0: 8%). - Reduced OS with locoregional therapies (HR: 2.6, *p* = 0.001). **ctDNA mutations post-treatment**—Detection prior to and 24 h after percutaneous ablation and persistence throughout the initial four cycles of atezolizumab + bevacizumab: lower OS and RFS.

AFP: alfa fetoprotein; AST: Aspartate Aminotransferase; AUC: area under the curve; BCLC: Barcelona Clinic Liver Cancer; CHIP: Clonal Hematopoiesis of Indeterminate Potential; CI: confidence interval; CNV: copy number variation; ddPCR: Droplet Digital PCR; EHR: extrahepatic recurrence; HBV: hepatitis B virus; HR: Hazard Ratio; HRG: high-risk gene; ICIs: immune checkpoint inhibitors; I-score: genomic instability score; LD-WGS: low-dose whole-genome sequencing; mos: months; MTA: Molecular Targeted Agent; NFE2L2: Nuclear Factor, Erythroid 2-Like 2; NGS: next-generation sequencing; OS: overall survival; PFS: progression-free survival; RFA: radiofrequency ablation; RFS: recurrence-free survival; RT: radiotherapy; TACE: transarterial chemoembolization; TERT: telomerase reverse transcriptase; TFx: tumor fraction; TKIs: Tyrosine Kinase Inhibitors; WGS: whole-genome sequencing.

Furthermore, another prospective cohort study from China evaluated the preoperative serum circulating ctDNA in a cohort of 258 patients undergoing curative HCC resection [60]. The number of mutant genes identified in ctDNA was significantly correlated with early tumor recurrence (HR: 2.2, *p* < 0.001). A high-risk gene (HRG) set, including mutations in APC, ARID1A, CDKN2A, FAT1, LRP1B, MAP3K1, PREX2, TERT and TP53, was identified, enabling risk stratification into low-, medium- and high-risk groups. High-risk individuals, especially those with solitary tumors, demonstrated significantly reduced RFS (HR: 13.0, *p* < 0.001). A nomogram integrating ctDNA-based risk levels and TNM staging accurately predicted recurrence (C-index: 0.76, 95% CI: 0.70–0.82). Notably, specific ctDNA mutations, such as FAT1 or LRP1B variants without TP53 mutations, predicted poor progression-free survival (PFS) in patients receiving lenvatinib combined with immune checkpoint inhibitors after recurrence (HR: 17.1, *p* < 0.001).

A fourth prospective case–control study from China assessed the efficacy of CNVs and tumor fraction (TFx) as biomarkers for predicting therapeutic response and prognosis in advanced HCC patients undergoing transarterial chemoembolization (TACE) [61]. Pre-TACE TFx was strongly correlated with tumor size (r = 0.563, *p* < 0.001), while patients with high TFx (≥0.1) had significantly shorter PFS [97 vs. 189 days (*p* = 0.002)] and OS [243 vs. 630 days (*p* < 0.001)]. Notably, fluctuations in TFx were predictive of TACE response, as patients exhibiting a TFx reduction exceeding 0.03 post-TACE experienced markedly improved outcomes compared to those with stable or rising TFx levels [PFS: 163 vs. 63 days; *p* = 0.007]. These TFx changes aligned with mRECIST assessments, highlighting their clinical utility for real-time treatment surveillance. Furthermore, specific CNVs, such as amplifications on chromosomes 1q, 6p, 8q, 10p and 18q, were linked to reduced lipiodol deposition (*p* = 0.008). Notably, chromosome 16q amplification and alterations in the NQO1 gene were linked to significantly shorter PFS (median: 83 vs. 158 days; *p* = 0.02).

In two prospective cohort studies from Japan, human telomerase reverse transcriptase (hTERT) promoter mutations were evaluated as predictors of the efficacy of TACE [15,62]. The rate of hTERT mutant cfDNA detection increased post-TACE (from 33% or 45% to 73% or 57%), reflecting increased tumor necrosis following therapy. Moreover, mutant cfDNA levels strongly correlated with tumor volume after TACE in one study (r^2^ = 0.449, *p* < 0.001) [15], highlighting its potential as a surrogate marker for tumor burden, whereas patients with reduced cfDNA levels following TACE demonstrated superior OS compared to those with consistently elevated cfDNA levels in the other study [62]. Notably, in both studies [15,62], cfDNA levels appeared to outperform traditional serum markers such as AFP, AFP-L3 and des-gamma-carboxy prothrombin (DCP) in accurately reflecting tumor burden and therapeutic response. In one study, which also included patients treated with RFA, post-RFA cfDNA levels and TERT promoter mutations also showed a significant increase (*p* < 0.001), reflecting tumor cell destruction caused by thermal ablation. Notably, patients exhibiting significant cfDNA increases following RFA showed improved treatment results, as the elevation was associated with successful tumor cell ablation.

Additionally, Li et al. examined the association between a tumor-specific mutation, namely the c.747 G>T mutation in the TP53 gene, in exosomal DNA and HCC prognosis. In a prospective cohort study, they analyzed samples from 60 HCC patients undergoing primary surgical treatment prior to hepatectomy for the detection of TP53 mutation status in cfDNA derived from exosomes. Their research revealed that patients with high-frequency TP53 mutations had worse recurrence-free survival (*p* < 0.01). High-frequency mutations were also correlated with microvascular invasion and poor prognosis (HR = 4.61; 95% CI: 1.71–12.48; *p* = 0.003) [63].

In a prospective cohort study from Korea including 37 advanced HCC patients treated with radiation therapy, the clinical utility of cfDNA was assessed as a biomarker to predict treatment outcomes by examining cfDNA genomic instability (I-score) [64]. Patients with a high baseline I-score (≥6.3) demonstrated significantly worse PFS (HR: 2.69, *p* = 0.017). Furthermore, the baseline I-score showed a strong positive correlation with tumor burden, quantified by the radiation therapy planning target volume.

In a prospective cohort study from France, the role of ctDNA as a prognostic biomarker across various therapeutic approaches was evaluated through a prospective observational cohort of 229 participants (173 with HCC and 56 controls) [56]. Using NGS on MiSeq and digital droplet PCR (ddPCR), ctDNA mutations were detected in plasma samples of 40% of active HCC cases, compared to 14.6% of patients with inactive HCC and only 1.8% of controls, with prevalence increasing in advanced disease stages (BCLC C: 65 vs. BCLC 0: 8%). Among patients treated with locoregional therapies, the presence of ctDNA mutations was independently linked to the risk of death (HR: 2.6, *p* = 0.001). Specifically, baseline mutations in ≥2 genes were linked to a higher risk of recurrence beyond the Milan criteria (75%) and extrahepatic spread (62.5%) compared to single or no mutation (*p* = 0.001). Moreover, ctDNA mutation detection both prior to and 24 h after percutaneous ablation was associated with the worst OS (*p* < 0.001) and RFS (*p* = 0.003).

Finally, in a prospective case–control study including HCC patients, the tumor mutational burden (TMB), which has often been reported to serve as a biomarker for immunotherapy response in various cancers [65], was shown to be higher in ctDNA than tissue samples and to correlate with genomic alterations in HTERT and TP53 [66].

### 6.2. Systemic Therapies (Table 7)

In a prospective study from Korea including 151 HCC patients treated with first-line sorafenib, elevated cfDNA concentration and higher I-score were significantly associated with worse treatment outcomes, including lower disease control rates, shorter time to progression (2.2 vs. 4.1 months) and reduced OS (4.1 vs. 14.8 months) [67].

**Table 7:** The main characteristics of studies evaluating the role of cell-free DNA (cfDNA) on response/prognosis after systemic therapy for hepatocellular carcinoma (HCC).

First Author, Year [Reference]	Study Population	Main Objective	Marker Type	Methodology	Key Findings
Oh, 2019 [67]	151 HCC patients receiving sorafenib	cfDNA levels, genome-wide CNAs, VEGFA amplification for prognosis post sorafenib	cfDNA levels, genome-wide CNAs (I-score) and VEGFA amplification	WGS of cfDNA; VEGFA analysis via ddPCR	**cfDNA**—Higher cfDNA linked to worse TTP (2.2 vs. 4.1 mos, HR: 1.71) and OS (4.1 vs. 14.8 mos, HR: 3.50). **Genome-Wide CNAs (I-score)**—Higher I-score: worse TTP (2.2 vs. 4.1 mos, HR: 2.09, *p* < 0.0001) and OS (4.6 vs. 14.8 mos, HR: 3.35). **VEGFA amplification**—VEGFA amplification levels: higher in HCC, but no significant correlation with treatment outcomes (DCR, TTP, or OS).
Mohamed, 2024 [68]	44 HCC patients receiving nivolumab	ctDNA as a biomarker for predicting OS and PFS	ctDNA alte-rations in TP53, PIK3CA, BRCA1, CCND1 and CTNNB1 genes	CLIA-certified Guardant360 platform targeting 74 cancer-related using NGS	**Mutation profiles**—PIK3CA and KIT mutations: associated with shorter PFS (*p* < 0.0004). CTNNB1 mutation: associated with longer PFS (*p* = 0.04). Mutations in PIK3CA, BRCA1 and CCND1 amplification: correlated with shorter OS (*p* < 0.0001, *p* < 0.0001 and *p* = 0.01, respectively).
Felden, 2020 [69]	51 HCC patients undergoing systemic therapy	ctDNA mutations as predictors of systemic therapy response	ctDNA alterations in TERT, TP53, CTNNB1, PI3K/MTOR pathway genes	Ultra-deep sequencing, digital droplet PCR	**Mutation profiles**—PI3K/MTOR mutations linked to TKI resistance (PFS: 2.1 vs. 3.7 mos, *p* < 0.001); WNT mutations not predictive of CPI response. Serial ctDNA profiling enabled treatment monitoring.

CCND1: Cyclin D1; CLIA: Clinical Laboratory Improvement Amendments; CNAs: Copy Number Alterations; CTNNB1: Catenin Beta 1; ddPCR: Droplet Digital Polymerase Chain Reaction; DCR: disease control rate; HR: Hazard Ratio; NGS: next-generation sequencing; OS: overall survival; PFS: progression-free survival; PIK3CA: Phosphatidylinositol-4,5-Bisphosphate 3-Kinase Catalytic Subunit Alpha; TTP: time to progression; VEGFA: Vascular Endothelial Growth Factor A; WGS: whole-genome sequencing; TERT: telomerase reverse transcriptase; TP53: Tumor Protein p53; MTOR: Mammalian Target of Rapamycin; TKI: Tyrosine Kinase Inhibitor; CPI: immune checkpoint inhibitor.

In the previously discussed French study [56], the persistence of ctDNA mutations during the initial four treatment cycles of atezolizumab plus bevacizumab therapy was found to be correlated with radiological progression (63.6%), while their clearance indicated a favorable response (*p* = 0.019). Lastly, new mutations, such as CTNNB1, emerged in some cases during disease progression, reflecting subclonal evolution.

Furthermore, in another prospective cohort study from the USA including 44 patients with advanced HCC who were treated with nivolumab, ctDNA profiling revealed somatic mutations in 93% of cases, with TP53 being the most frequently altered gene [68]. Mutations in PIK3CA, BRCA1 and CCND1 amplifications were significantly associated with shorter OS, whereas mutations in KIT and PIK3CA correlated with diminished PFS. Conversely, CTNNB1 mutations correlated with extended PFS.

Finally, another prospective study from the USA highlighted the utility of liquid biopsy in guiding personalized therapy for advanced HCC. Using ultra-deep sequencing and digital droplet PCR, this study identified frequent mutations in TERT promoter (51%), TP53 (32%) and CTNNB1 (17%). Notably, mutations in the PI3K/MTOR pathway were linked to primary resistance to TKIs, resulting in significantly shorter PFS (2.1 vs. 3.7 months, *p* < 0.001), while WNT pathway mutations did not predict response to immune checkpoint inhibitors. Serial ctDNA profiling demonstrated potential for real-time treatment monitoring, detecting emerging resistant clones and refining therapeutic strategies [69].

## 7. Discussion

Circulating cfDNA and its derivative ctDNA, which represent the main components of a liquid biopsy, are increasingly assessed as potential biomarkers for early diagnosis and prognosis of several cancers, including HCC, as well as for guiding treatment decisions [8,70]. The research in cfDNA markers is a rapidly evolving field in relation to both applied methodologies and diseases with potential clinical relevance [7]. Despite all the research efforts, several issues must be further clarified before cfDNA may be used in clinical practice, such as the standardization of optimal collection and centrifugation protocols, cfDNA isolation and quantification, methods for the detection of genomic alterations or specific mutations and others [8,70]. At the same time, the number of studies using cfDNA markers and cfDNA data have been increasing in recent years suggesting that cfDNA markers could represent reliable tools for the optimization of early cancer diagnosis and management [8,70].

In early studies in HCC, levels of circulating cfDNA were shown to be higher in HCC patients than patients with chronic liver diseases [18,19,21], but their association with the severity of inflammation was considered to represent a major limitation [19,71]. Thus, research focused on ctDNA, which however is estimated to be <1% of the total cfDNA [72], and the species of cfDNA. In particular, cfDNA integrity, usually determined by Alu 247/Alu 115 ratio, was reported to increase in the serum of HCC patients in some [10,21] but not all studies [24,26]. Mitochondrial DNA has also been shown to be higher in HCC patients, but it has been assessed in only one study [23].

Numerous studies have focused on the evaluation of cfDNA and especially ctDNA methylation and mutations as biomarkers for early HCC diagnosis, as there are characteristic methylation changes in tumor DNA, which usually develop early in carcinogenesis and are expressed in ctDNA [73]. Thus, several ctDNA methylation markers have been repeatedly shown to offer excellent diagnostic accuracy (AUROCs >0.90) with high rates of sensitivity (60–94%) and specificity (91–98.5%) for HCC diagnosis and, more importantly, for early HCC diagnosis [27,28,29,30,31,32,33]. Additional efforts, including a methylation panel established by NGS [34], the HelioLiver Test (combined cfDNA methylation patterns with clinical characteristics and protein tumor markers) [35] and hypermethylation of specific cfDNA regions [36,37,38,39,40,41,42,43], have also been shown to offer excellent diagnostic accuracy for HCC diagnosis. In several studies, the combined use of ctDNA methylation markers and AFP was shown to improve the diagnostic performance and especially sensitivity [28,30,34,36,37,38,40,42].

cfDNA fragmentation patterns and circulating nucleosomes have also been assessed as potential diagnostic biomarkers for HCC. In several studies, cfDNA fragmentation profiles have always been shown to offer high rates of sensitivity (75–97%) and specificity (80–99%) for the detection of HCC [13,46,47,48,49,50]. Moreover, specific scores, including cfDNA fragmentation markers, motif scores and nucleosomes with or without the combination with established HCC markers and clinicoepidemiological scores, were also shown to offer excellent diagnostic accuracy for HCC diagnosis [51,52,53]. Finally, the role of cfDNA mutations sometimes combined with established HCC serum markers has been evaluated in a limited number of studies [54,55]. It is of interest that extracellular vesicles containing DNA have been shown to play a crucial role in liver fibrosis mediating inflammation, which may lead to liver carcinogenesis [74].

The role of circulating cfDNA markers and in particular of ctDNA on the prognosis of untreated patients with HCC has been assessed in only two studies providing promising results for the prediction of survival [22,27]. However, more data are required on this topic, although it may be currently unethical to keep diagnosed HCC patients under no treatment.

On the other hand, the predictive role of circulating cfDNA levels or mutations of ctDNA, as well as CNV indicators, has been confirmed in several studies including patients with HCC undergoing liver resection [20,58,59,60]. The detection of any such marker post-hepatectomy seems to reflect the minimum residual disease and thus a high risk for HCC recurrence, which can guide postoperative monitoring and treatment strategies. Moreover, the detection of these markers, which may signify the metastatic capability of HCC, appears to be associated with the increased probability of distant metastasis and reduced overall survival, suggesting that these patients could benefit from enhanced monitoring and perhaps from adjuvant therapy.

Similarly to HCC patients undergoing hepatectomy, in patients with advanced HCC undergoing TACE, post-TACE cfDNA and ctDNA levels and pre-TACE CNVs and TFx have been shown to predict therapeutic response and survival [15,61,62], which seems to be favorably associated with reduced cfDNA levels and increasing rates of hTERT promoter mutations [15,56,62]. The extent of cfDNA alterations and ctDNA mutations during RFA have also been suggested as useful non-invasive predictors of treatment effectiveness in HCC patients treated with RFA potentially guiding management decisions [56,62]. Moreover, cfDNA genomic instability was shown to predict treatment outcomes in HCC patients undergoing radiotherapy [64].

In the few available studies including HCC patients treated with systemic therapies, the elevated cfDNA concentration and persistence of ctDNA mutations have been associated with worse outcomes [56,67,68]. Such findings, if further validated, might lead to the advancement of personalized treatment strategies of patients with advanced HCC.

## 8. Conclusions

The utilization of cfDNA is of great importance in the field of HCC, as cfDNA markers may not only be used for screening and early diagnosis, but also for evaluation of the aggressiveness, post-hepatectomy detection of recurrence and investigation of resistance to anti-cancer therapies. There are many data showing that cfDNA species and especially ctDNA methylation markers, as well as cfDNA fragmentation patterns and circulating nucleosomes, could be rather useful liquid biomarkers for early HCC diagnosis, although further research and methodological improvements are necessary for the determination of the most precise and practical non-invasive HCC biomarker and for widespread use of such markers in clinical practice. On the other hand, given the low sensitivity of currently used biomarkers and methods for HCC surveillance, such as AFP and ultrasonography with wide inter-observer variation, more precise HCC biomarkers are certainly required. In addition to HCC surveillance, cfDNA markers can be extremely useful for monitoring treatment effectiveness and for early detection of minimal residual disease post-treatment, thus optimizing patient management by potentially guiding additional therapeutic interventions that could improve patient outcomes. However, further research is still needed in several areas, including the standardization of methodologies, the sensitivity of ctDNA detection and the validation of such biomarkers in appropriate large cohorts. Most cfDNA-related trials for HCC are phase II and occasionally III, targeting early detection, prognosis and treatment monitoring. No cfDNA-based assay for HCC has received full FDA/EMA approval, but some have been granted breakthrough device designation by the FDA. Therefore, future large-scale validation trials and regulatory submissions are needed to implement cfDNA liquid biopsies in routine clinical practice.

## Figures and Tables

**Table 1:** Circulating cell-free DNA (cfDNA) concentration and integrity for diagnosis of hepatocellular carcinoma (HCC).

First Author, Year [Reference]	HCC Patients	Controls	Species of cfDNA	Sensitivity	Specificity	AUROC (95% CI)	Other Key Findings
Izuka, 2006 [18]	52 HCV	30 HCV and 18 controls	cfDNA levels measured by rtPCR (GSTP1 gene)	69%	93%	0.90 (0.83–0.96)	cfDNA superior than AFP or PIVKA-II
Iida, 2008 [19]	96 HCV	100 HCV	Serum cfDNA levels	NA	NA	NA	cfDNA levels: higher in HCC than non-HCC cases, *p* < 0.001; High cfDNA level: association with HCC inflammatory status
Tokuhisa, 2007 [20]	96 HCV	100 HCV	cfDNA levels	NA	NA	NA	cfDNA higher in HCC vs. non-HCC pts (116 vs. 34 ng/mL, *p* < 0.0001)
Elzehery, 2022 [21]	50 HCV	50 HCV LC and 50 controls	cfDNA levels and cfDNA integrity (Alu247/115)	cfDNA: 82% integrity: 70%	cfDNA: 76% integrity: 88%	cfDNA: 0.83 (0.75–0.91); integrity: 0.86 (0.78–0.93)	
Lian, 2024 [22]	63 HBV	90 CHB	Genome-wide copy number and tumor content in cfDNA	1 year pre-diagnosis 23% BCLC A: 30%	98%	NA	High tumor content associated with tumor stage and poor survival
Jiang, 2015 [23]	90	135 controls (103 CLD)	ctDNA size and mitochondrial DNA	Mitochondrial DNA: 80%	Mitochondrial DNA: 94%	Mitochondrial DNA: 0.93	
Huang, 2016 [24]	53 (and 19 non-HCC cancers)	37 controls	cfDNA integrity: Alu247/Alu115	43%	100%	0.705	cfDNA integrity: may be useful for HCC treatment surveillance
Papatheodoridi, 2021 [10]	19 CHB	38 CHB	cfDNA, Alu115, Alu247, nucleosomes and cfDNA integrity (Alu247/115)	NA	NA	NA	HCC-CHB vs. CHB—median Alu 247: 64 vs. 23, *p* = 0.010; Alu247/115: 1 vs. 0.7, *p* < 0.001
Papatheodoridi, 2021 [25]	37 CHB	74 CHB	cfDNA levels, Alu 247 and 115, RNase P coding DNA, mitochondrial DNA, DNA methylation	NA	NA	0.80 (0.71–0.89) for RNase P levels	Median Alu247: 123 vs. 69, *p* = 0.042; median RNase P: GE 68 vs. 15, *p* < 0.001
Kamal, 2022 [26]	80 HCV	80 HCV LC	cfDNA integrity (Alu115/247) by rtPCR	85%	97.5%	NA	

AFP: alfa fetoprotein; AUROC: area under receiving operating characteristic; CHB: chronic hepatitis B; CI: confidence interval; CLD: chronic liver disease; HBV: hepatitis B virus; HCV: hepatitis C virus; LC: liver cirrhosis; NA: not available; rtPCR: reverse transcription polymerase chain reaction.

**Table 5:** Main characteristics of studies evaluating cell-free DNA (cfDNA) as a marker of prognosis of hepatocellular carcinoma (HCC).

First Author, Year [Reference]	Study Population	Main Objective	Marker Type	Methodology	Key Findings
Xu, 2017 [27]	1098 HCC patients and 835 healthy controls	ctDNA methylation markers for HCC diagnosis, treatment response and prognosis	ctDNA methyla-tion markers	Bisulfite sequencing, padlock probe capture, LASSO and random-forest feature selection	**Prognostic model**—eight-marker panel correlated with survival outcomes; high-risk (cp-score > −0.24): worse survival than low-risk patients **Treatment response monitoring**—decreased cp-scores post-treatment: better outcomes than rising scores, which correlated with tumor burden and progression.
Lian, 2024 [22]	67 patients with HBV-related HCC and 90 controls	Tumor-derived cfDNA (tumor content) as biomarker for monitoring, HCC progression and prognosis	cfDNA tumor content	Shallow WGS and ichorCNA method to assess genome-wide copy number variations and tumor content in cfDNA	**Tumor content and stage/survival**—high tumor content in cfDNA correlated with advanced tumor stage (*p* < 0.001) and poorer survival (HR: 12.3, 95% CI: 1.4–107.9; *p* = 0.023) **Post-treatment monitoring:** tumor content turned negative post-surgery *p* = 0.027) but remained positive after TACE (*p* = 0.578).

CI: confidence interval; ctDNA: circulating tumor DNA; HR: Hazard Ratio; TACE: transarterial chemoembolization; WGS: whole-genome sequencing.

## References

[1] Bray F., Ferlay J., Soerjomataram I., Siegel R.L., Torre L.A., Jemal A. (2018). Global cancer statistics 2018: GLOBOCAN estimates of incidence and mortality worldwide for 36 cancers in 185 countries. CA Cancer J. Clin..

[2] McGlynn K.A., Petrick J.L., El-Serag H.B. (2021). Epidemiology of Hepatocellular Carcinoma. Hepatology.

[3] Villanueva A. (2019). Hepatocellular Carcinoma. N. Engl. J. Med..

[4] EASL (2018). EASL Clinical Practice Guidelines: Management of hepatocellular carcinoma. J. Hepatol..

[5] Llovet J.M., Kelley R., Villanueva A., Singal A., Pikarsky E., Roayaie S., Lencioni R., Koike K., Zucman-Rossi J., Finn R.S. (2021). Hepatocellular carcinoma. Nat. Rev. Dis. Primers.

[6] Lehrich B.M., Zhang J., Monga S.P., Dhanasekaran R. (2024). Battle of the biopsies: Role of tissue and liquid biopsy in hepatocellular carcinoma. J. Hepatol..

[7] Schwarzenbach H., Hoon D.S., Pantel K. (2011). Cell-free nucleic acids as biomarkers in cancer patients. Nat. Rev. Cancer.

[8] Sasaki R., Kanda T., Yokosuka O., Kato N., Matsuoka S., Moriyama M. (2019). Exosomes and Hepatocellular Carcinoma: From Bench to Bedside. Int. J. Mol. Sci..

[9] Chan Y.T., Zhang C., Wu J., Lu P., Xu L., Yuan H., Feng Y., Chen Z.S., Wang N. (2024). Biomarkers for diagnosis and therapeutic options in hepatocellular carcinoma. Mol. Cancer.

[10] Papatheodoridi A., Karakousis N., Lembessis P., Chatzigeorgiou A., Papatheodoridis G.V. (2021). The Significance of Circulating Cell-Free DNA Markers in Chronic Hepatitis B Patients with Hepatocellular Carcinoma. Pathogens.

[11] Yan L., Chen Y., Zhou J., Zhao H., Zhang H., Wang G. (2018). Diagnostic value of circulating cell-free DNA levels for hepatocellular carcinoma. Int. J. Infect. Dis..

[12] Tran N.H., Kisiel J., Roberts L.R. (2021). Using cell-free DNA for HCC surveillance and prognosis. JHEP Rep..

[13] Sun X., Feng W., Cui P., Ruan R., Ma W., Han Z., Sun J., Pan Y., Zhu J., Zhong X. (2022). Detection and monitoring of HBV-related hepatocellular carcinoma from plasma cfDNA fragmentation profiles. Genomics.

[14] Kaseb A.O., Sanchez N.S., Sen S., Kelley R.K., Tan B., Bocobo A.G., Lim K.H., Abdel-Wahab R., Uemura M., Pestana R.C. (2019). Molecular Profiling of Hepatocellular Carcinoma Using Circulating Cell-Free DNA. Clin. Cancer Res..

[15] Muraoka M., Maekawa S., Katoh R., Komiyama Y., Nakakuki N., Takada H., Matsuda S., Suzuki Y., Sato M., Tatsumi A. (2021). Usefulness of Cell-Free Human Telomerase Reverse Transcriptase Mutant DNA Quantification in Blood for Predicting Hepatocellular Carcinoma Treatment Efficacy. Hepatol. Commun..

[16] Yang J., Lin N., Niu M., Yin B. (2024). Circulating tumor DNA mutation analysis: Advances in its application for early diagnosis of hepatocellular carcinoma and therapeutic efficacy monitoring. Aging.

[17] Banini B.A., Sanyal A.J. (2019). The use of cell free DNA in the diagnosis of HCC. Hepatoma Res..

[18] Iizuka N., Sakaida I., Moribe T., Fujita N., Miura T., Stark M., Tamatsukuri S., Ishitsuka H., Uchida K., Terai S. (2006). Elevated levels of circulating cell-free DNA in the blood of patients with hepatitis C virus-associated hepatocellular carcinoma. Anticancer. Res..

[19] Iida M., Iizuka N., Sakaida I., Moribe T., Fujita N., Miura T., Tamatsukuri S., Ishitsuka H., Uchida K., Terai S. (2008). Relation between serum levels of cell-free DNA and inflammation status in hepatitis C virus-related hepatocellular carcinoma. Oncol. Rep..

[20] Tokuhisa Y., Iizuka N., Sakaida I., Moribe T., Fujita N., Miura T., Tamatsukuri S., Ishitsuka H., Uchida K., Terai S. (2007). Circulating cell-free DNA as a predictive marker for distant metastasis of hepatitis C virus-related hepatocellular carcinoma. Br. J. Cancer.

[21] Elzehery R., Effat N., El Farahaty R., Elsayed Farag R., Abo-Hashem E.M., Elhelaly R. (2022). Circulating Cell-Free DNA and DNA Integrity as Molecular Diagnostic Tools in Hepatocellular Carcinoma. Am. J. Clin. Pathol..

[22] Lian S., Lu C., Li F., Yu X., Ai L., Wu B., Gong X., Zhou W., Liang X., Zhan J. (2024). Monitoring Hepatocellular Carcinoma Using Tumor Content in Circulating Cell-Free DNA. Clin. Cancer Res..

[23] Jiang P., Chan C.W., Chan K.C., Cheng S.H., Wong J., Wong V.W., Wong G.L., Chan S.L., Mok T.S., Chan H.L. (2015). Lengthening and shortening of plasma DNA in hepatocellular carcinoma patients. Proc. Natl. Acad. Sci. USA.

[24] Huang A., Zhang X., Zhou S.L., Cao Y., Huang X.W., Fan J., Yang X.R., Zhou J. (2016). Plasma Circulating Cell-free DNA Integrity as a Promising Biomarker for Diagnosis and Surveillance in Patients with Hepatocellular Carcinoma. J. Cancer.

[25] Papatheodoridi A., Chatzigeorgiou A., Chrysavgis L., Lembessis P., Loglio A., Facchetti F., Cholongitas E., Koutsilieris M., Lampertico P., Papatheodoridis G. (2021). Circulating cell-free DNA species affect the risk of hepatocellular carcinoma in treated chronic hepatitis B patients. J. Viral Hepat..

[26] Kamal M.M., Abdelaziz A.O., El-Baz H.N., Mohamed G.M., Saleh S.S., Nabeel M.M., Elbaz T.M., Lithy R., Shousha H.I. (2022). Plasma cell-free DNA integrity index and hepatocellular carcinoma treated or not with direct-acting antivirals: A case-control study. Arab. J. Gastroenterol..

[27] Xu R.-h., Wei W., Krawczyk M., Wang W., Luo H., Flagg K., Yi S., Shi W., Quan Q., Li K. (2017). Circulating tumour DNA methylation markers for diagnosis and prognosis of hepatocellular carcinoma. Nat. Mater..

[28] Luo B., Ma F., Liu H., Hu J., Rao L., Liu C., Jiang Y., Kuangzeng S., Lin X., Wang C. (2022). Cell-free DNA methylation markers for differential diagnosis of hepatocellular carcinoma. BMC Med..

[29] Wang P., Song Q., Ren J., Zhang W., Wang Y., Zhou L., Wang D., Chen K., Jiang L., Zhang B. (2022). Simultaneous analysis of mutations and methylations in circulating cell-free DNA for hepatocellular carcinoma detection. Sci. Transl. Med..

[30] Phan T.H., Chi Nguyen V.T., Thi Pham T.T., Nguyen V.C., Ho T.D., Quynh Pham T.M., Tran T.H., Nguyen T.D., Khang Le N.D., Nguyen T.H. (2022). Circulating DNA methylation profile improves the accuracy of serum biomarkers for the detection of nonmetastatic hepatocellular carcinoma. Future Oncol..

[31] Deng Z., Ji Y., Han B., Tan Z., Ren Y., Gao J., Chen N., Ma C., Zhang Y., Yao Y. (2023). Early detection of hepatocellular carcinoma via no end-repair enzymatic methylation sequencing of cell-free DNA and pre-trained neural network. Genome Med..

[32] Guo P., Zheng H., Li Y., Li Y., Xiao Y., Zheng J., Zhu X., Xu H., He Z., Zhang Q. (2023). Hepatocellular carcinoma detection via targeted enzymatic methyl sequencing of plasma cell-free DNA. Clin. Epigenetics.

[33] Wang J., Yang L., Diao Y., Liu J., Li J., Li R., Zheng L., Zhang K., Ma Y., Hao X. (2021). Circulating tumour DNA methylation in hepatocellular carcinoma diagnosis using digital droplet PCR. J. Int. Med. Res..

[34] Lewin J., Kottwitz D., Aoyama J., deVos T., Garces J., Hasinger O., Kasielke S., Knaust F., Rathi P., Rausch S. (2021). Plasma cell free DNA methylation markers for hepatocellular carcinoma surveillance in patients with cirrhosis: A case control study. BMC Gastroenterol..

[35] Lin N., Lin Y., Xu J., Liu D., Li D., Meng H., Gallant M.A., Kubota N., Roy D., Li J.S. (2022). A multi-analyte cell-free DNA-based blood test for early detection of hepatocellular carcinoma. Hepatol. Commun..

[36] Han L.Y., Fan Y.C., Mu N.N., Gao S., Li F., Ji X.F., Dou C.Y., Wang K. (2014). Aberrant DNA methylation of G-protein-coupled bile acid receptor Gpbar1 (TGR5) is a potential biomarker for hepatitis B Virus associated hepatocellular carcinoma. Int. J. Med. Sci..

[37] Li F., Fan Y.C., Gao S., Sun F.K., Yang Y., Wang K. (2014). Methylation of serum insulin-like growth factor-binding protein 7 promoter in hepatitis B virus-associated hepatocellular carcinoma. Genes Chromosom. Cancer.

[38] Huang G., Krocker J.D., Kirk J.L., Merwat S.N., Ju H., Soloway R.D., Wieck L.R., Li A., Okorodudu A.O., Petersen J.R. (2014). Evaluation of INK4A promoter methylation using pyrosequencing and circulating cell-free DNA from patients with hepatocellular carcinoma. Clin. Chem. Lab. Med..

[39] Oussalah A., Rischer S., Bensenane M., Conroy G., Filhine-Tresarrieu P., Debard R., Forest-Tramoy D., Josse T., Reinicke D., Garcia M. (2018). Plasma mSEPT9: A Novel Circulating Cell-free DNA-Based Epigenetic Biomarker to Diagnose Hepatocellular Carcinoma. EBioMedicine.

[40] Kim S.C., Kim D.W., Cho E.J., Lee J.Y., Kim J., Kwon C., Kim-Ha J., Hong S.K., Choi Y., Yi N.J. (2023). A circulating cell-free DNA methylation signature for the detection of hepatocellular carcinoma. Mol. Cancer.

[41] Cai J., Chen L., Zhang Z., Zhang X., Lu X., Liu W., Shi G., Ge Y., Gao P., Yang Y. (2019). Genome-wide mapping of 5-hydroxymethylcytosines in circulating cell-free DNA as a non-invasive approach for early detection of hepatocellular carcinoma. Gut.

[42] Cai Z., Zhang J., He Y., Xia L., Dong X., Chen G., Zhou Y., Hu X., Zhong S., Wang Y. (2021). Liquid biopsy by combining 5-hydroxymethylcytosine signatures of plasma cell-free DNA and protein biomarkers for diagnosis and prognosis of hepatocellular carcinoma. ESMO Open.

[43] Guo D.Z., Huang A., Wang Y.C., Zhou S., Wang H., Xing X.L., Zhang S.Y., Cheng J.W., Xie K.H., Yang Q.C. (2024). Early detection and prognosis evaluation for hepatocellular carcinoma by circulating tumour DNA methylation: A multicentre cohort study. Clin. Transl. Med..

[44] Kim K., Zheng Y., Joyce B.T., Nannini D.R., Wang J., Qu Y., Hawkins C.A., Okeke E., Lesi O.A., Roberts L.R. (2024). Cell-free DNA methylation-based inflammation score as a marker for hepatocellular carcinoma among people living with HIV. Hepatol. Int..

[45] Manea I., Iacob R., Iacob S., Cerban R., Dima S., Oniscu G., Popescu I., Gheorghe L. (2023). Liquid biopsy for early detection of hepatocellular carcinoma. Front. Med..

[46] Jin C., Liu X., Zheng W., Su L., Liu Y., Guo X., Gu X., Li H., Xu B., Wang G. (2021). Characterization of fragment sizes, copy number aberrations and 4-mer end motifs in cell-free DNA of hepatocellular carcinoma for enhanced liquid biopsy-based cancer detection. Mol. Oncol..

[47] Zhang X., Wang Z., Tang W., Wang X., Liu R., Bao H., Chen X., Wei Y., Wu S., Bao H. (2022). Ultrasensitive and affordable assay for early detection of primary liver cancer using plasma cell-free DNA fragmentomics. Hepatology.

[48] Meng Z., Ren Q., Zhong G., Li S., Chen Y., Wu W., Feng Y., Mao M., Zhang F., Long G. (2021). Noninvasive Detection of Hepatocellular Carcinoma with Circulating Tumor DNA Features and α-Fetoprotein. J. Mol. Diagn..

[49] Foda Z.H., Annapragada A.V., Boyapati K., Bruhm D.C., Vulpescu N.A., Medina J.E., Mathios D., Cristiano S., Niknafs N., Luu H.T. (2023). Detecting Liver Cancer Using Cell-Free DNA Fragmentomes. Cancer Discov..

[50] Nguyen V.C., Nguyen T.H., Phan T.H., Tran T.T., Pham T.T.T., Ho T.D., Nguyen H.H.T., Duong M.L., Nguyen C.M., Nguyen Q.B. (2023). Fragment length profiles of cancer mutations enhance detection of circulating tumor DNA in patients with early-stage hepatocellular carcinoma. BMC Cancer.

[51] Chen L., Abou-Alfa G.K., Zheng B., Liu J.F., Bai J., Du L.T., Qian Y.S., Fan R., Liu X.L., Wu L. (2021). Genome-scale profiling of circulating cell-free DNA signatures for early detection of hepatocellular carcinoma in cirrhotic patients. Cell Res..

[52] Fan R., Chen L., Zhao S., Yang H., Li Z., Qian Y., Ma H., Liu X., Wang C., Liang X. (2023). Novel, high accuracy models for hepatocellular carcinoma prediction based on longitudinal data and cell-free DNA signatures. J. Hepatol..

[53] Chen L., Wu T., Fan R., Qian Y.S., Liu J.F., Bai J., Zheng B., Liu X.L., Zheng D., Du L.T. (2024). Cell-free DNA testing for early hepatocellular carcinoma surveillance. EBioMedicine.

[54] Wu T., Fan R., Bai J., Yang Z., Qian Y.-S., Du L.-T., Wang C.-Y., Wang Y.-C., Jiang G.-Q., Zheng D. (2023). The development of a cSMART-based integrated model for hepatocellular carcinoma diagnosis. J. Hematol. Oncol..

[55] Li C.L., Ho M.C., Lin Y.Y., Tzeng S.T., Chen Y.J., Pai H.Y., Wang Y.C., Chen C.L., Lee Y.H., Chen D.S. (2020). Cell-Free Virus-Host Chimera DNA from Hepatitis B Virus Integration Sites as a Circulating Biomarker of Hepatocellular Cancer. Hepatology.

[56] Campani C., Imbeaud S., Couchy G., Ziol M., Hirsch T.Z., Rebouissou S., Noblet B., Nahon P., Hormigos K., Sidali S. (2024). Circulating tumour DNA in patients with hepatocellular carcinoma across tumour stages and treatments. Gut.

[57] Choi E.J., Kim Y.J. (2022). Liquid biopsy for early detection and therapeutic monitoring of hepatocellular carcinoma. J. Liver Cancer.

[58] Long G., Fang T., Su W., Mi X., Zhou L. (2020). The prognostic value of postoperative circulating cell-free DNA in operable hepatocellular carcinoma. Scand. J. Gastroenterol..

[59] Wang Y., Zhou K., Wang X., Liu Y., Guo D., Bian Z., Su L., Liu K., Gu X., Guo X. (2021). Multiple-level copy number variations in cell-free DNA for prognostic prediction of HCC with radical treatments. Cancer Sci..

[60] Fu Y., Yang Z., Hu Z., Yang Z., Pan Y., Chen J., Wang J., Hu D., Zhou Z., Xu L. (2022). Preoperative serum ctDNA predicts early hepatocellular carcinoma recurrence and response to systemic therapies. Hepatol. Int..

[61] Dong X., Chen G., Huang X., Li Z., Peng F., Chen H., Zhou Y., He L., Qiu L., Cai Z. (2022). Copy number profiling of circulating free DNA predicts transarterial chemoembolization response in advanced hepatocellular carcinoma. Mol. Oncol..

[62] Nakatsuka T., Nakagawa H., Hayata Y., Wake T., Yamada T., Nishibatake Kinoshita M., Nakagomi R., Sato M., Minami T., Uchino K. (2021). Post-treatment cell-free DNA as a predictive biomarker in molecular-targeted therapy of hepatocellular carcinoma. J. Gastroenterol..

[63] Li Y., Wu J., Li E., Xiao Z., Lei J., Zhou F., Yin X., Hu D., Mao Y., Wu L. (2022). TP53 mutation detected in circulating exosomal DNA is associated with prognosis of patients with hepatocellular carcinoma. Cancer Biol. Ther..

[64] Kim D.Y., Cho E.H., Kim J.S., Chie E.K., Kang H.C. (2023). Plasma Circulating Cell-free DNA in Advanced Hepatocellular Carcinoma Patients Treated with Radiation Therapy. Vivo.

[65] Marabelle A., Fakih M., Lopez J., Shah M., Shapira-Frommer R., Nakagawa K., Chung H.C., Kindler H.L., Lopez-Martin J.A., Miller W.H. (2020). Association of tumour mutational burden with outcomes in patients with advanced solid tumours treated with pembrolizumab: Prospective biomarker analysis of the multicohort, open-label, phase 2 KEYNOTE-158 study. Lancet Oncol..

[66] Franses J.W., Lim M., Burgoyne A.M., Mody K., Lennerz J., Chang J., Imperial R., Dybel S.N., Dinh T.M., Masannat J. (2022). Profile and Predictors of Blood Tumor Mutational Burden in Advanced Hepatocellular Carcinoma. Oncologist.

[67] Oh C.R., Kong S.Y., Im H.S., Kim H.J., Kim M.K., Yoon K.A., Cho E.H., Jang J.H., Lee J., Kang J. (2019). Genome-wide copy number alteration and VEGFA amplification of circulating cell-free DNA as a biomarker in advanced hepatocellular carcinoma patients treated with Sorafenib. BMC Cancer.

[68] Mohamed Y.I., Lee S.S., Demir T., Chamseddine S., Hu Z.I., Xiao L., Elsayes K., Morris J.S., Wolff R.A., Hiatia R. (2024). Circulating tumor DNA (ctDNA) as a biomarker of response to therapy in advanced Hepatocellular carcinoma treated with Nivolumab. Cancer Biomark..

[69] von Felden J., Craig A.J., Garcia-Lezana T., Labgaa I., Haber P.K., D’Avola D., Asgharpour A., Dieterich D., Bonaccorso A., Torres-Martin M. (2021). Mutations in circulating tumor DNA predict primary resistance to systemic therapies in advanced hepatocellular carcinoma. Oncogene.

[70] Cisneros-Villanueva M., Hidalgo-Pérez L., Rios-Romero M., Cedro-Tanda A., Ruiz-Villavicencio C.A., Page K., Hastings R., Fernandez-Garcia D., Allsopp R., Fonseca-Montaño M.A. (2022). Cell-free DNA analysis in current cancer clinical trials: A review. Br. J. Cancer.

[71] Kustanovich A., Schwartz R., Peretz T., Grinshpun A. (2019). Life and death of circulating cell-free DNA. Cancer Biol. Ther..

[72] Lyu X., Tsui Y.M., Ho D.W., Ng I.O. (2022). Liquid Biopsy Using Cell-Free or Circulating Tumor DNA in the Management of Hepatocellular Carcinoma. Cell Mol. Gastroenterol. Hepatol..

[73] Warton K., Mahon K.L., Samimi G. (2016). Methylated circulating tumor DNA in blood: Power in cancer prognosis and response. Endocr. Relat. Cancer.

[74] Li J., Yuan Y., Fu Q., Chen M., Liang H., Chen X., Long X., Zhang B., Zhao J., Chen Q. (2024). Novel insights into the role of immunomodulatory extracellular vesicles in the pathogenesis of liver fibrosis. Biomark. Res..

